# A review of phytochemistry, metabolite changes, and medicinal uses of the common food mung bean and its sprouts (*Vigna radiata*)

**DOI:** 10.1186/1752-153X-8-4

**Published:** 2014-01-17

**Authors:** Dongyan Tang, Yinmao Dong, Hankun Ren, Li Li, Congfen He

**Affiliations:** 1 Department of Chemistry, Harbin Institute of Technology, Harbin 150000, China; 2 Beijing Key Lab of Plant Resources Research and Development, Beijing Technology and Business University, Beijing 100048, China

**Keywords:** Mung bean, Nutritional value, Chemical constituents, Metabolites, Biological activities

## Abstract

The seeds and sprouts of mung bean (*Vigna radiata*), a common food, contain abundant nutrients with biological activities. This review provides insight into the nutritional value of mung beans and its sprouts, discussing chemical constituents that have been isolated in the past few decades, such as flavonoids, phenolic acids, organic acids, amino acids, carbohydrates, and lipids. Moreover, we also summarize dynamic changes in metabolites during the sprouting process and related biological activities, including antioxidant, antimicrobial, anti-inflammatory, antidiabetic, antihypertensive, lipid metabolism accommodation, antihypertensive, and antitumor effects, etc., with the goal of providing scientific evidence for better application of this commonly used food as a medicine.

## Review

### Introduction

With increasing clinical evidence suggesting that plant-derived foods have various potential health benefits, their consumption has been growing at a rate of 5%-10% per year [1]. Moreover, many worldwide health organizations have recommended an increase in the intake of plant-derived foods to improve health status and prevent chronic diseases [2].

The mung bean (*Vigna radiata*) has been consumed as a common food in China for more than 2,000 years. It is well known for its detoxification activities and is used to refresh mentality, alleviate heat stroke, and reduce swelling in the summer. In the book Ben Cao Qiu Zhen (本草求真), the mung bean was recorded to be beneficial in the regulation of gastrointestinal upset and to moisturize the skin [3]. The seeds and sprouts of mung beans are also widely used as a fresh salad vegetable or common food in India, Bangladesh, South East Asia, and western countries [4]. As a food, mung beans contain balanced nutrients, including protein and dietary fiber, and significant amounts of bioactive phytochemicals. High levels of proteins, amino acids, oligosaccharides, and polyphenols in mung beans are thought to be the main contributors to the antioxidant, antimicrobial, anti-inflammatory, and antitumor activities of this food and are involved in the regulation of lipid metabolism [5-8].

In recent years, studies have shown that the sprouts of mung beans after germination have more obvious biological activities and more plentiful secondary metabolites since relevant biosynthetic enzymes are activated during the initial stages of germination. Thus, germination is thought to improve the nutritional and medicinal qualities of mung beans [9]. Highly efficient use of mung beans according to evidence demonstrated from scientific experiments will be beneficial to the application of mung beans as a health food, medicine, and cosmetic [10]. In the present review, we summarize the nutritional value, chemical constituents, and metabolite changes during the sprouting process, as well as pharmacological activities, and clinical applications of mung beans, which will provide a better understanding of the potential applications of this common food.

### Nutritional value of mung beans as a common food

Mung beans are a pulse or food legume crop used primarily as dried seeds and occasionally as forage or green pods and seeds for vegetables [11]. Dried seeds may be eaten whole or split, cooked, fermented, or milled and ground into flour. Mung beans can also be made into products like soups, porridge, confections, curries, and alcoholic beverages. In western cultures, mung bean sprouts are popularly used as a fresh salad vegetable [12].

Importantly, mung beans are composed of about 20%–24% protein. Globulin and albumin are the main storage proteins found in mung bean seeds and make up over 60% and 25% of the total mung bean protein, respectively. Therefore, due to its high protein content and digestibility, consumption of mung beans in combination with cereals can significantly increase the quality of protein in a meal [13,14]. Mung bean protein is rich in essential amino acids, such as total aromatic amino acids, leucine, isoleucine, and valine, as compared with the FAO/WHO (1973) reference. However, compared with the reference pattern, mung bean protein is slightly deficient in threonine, total sulfur amino acids, lysine, and tryptophan [15]. Moreover, the proteolytic cleavage of proteins during sprouting leads to a significant increase in the levels of amino acids.

Mung beans have much greater carbohydrate content (50%–60%) than soybeans, and starch is the predominant carbohydrate in the legume. Due to its high starch content, mung beans have typically been used for the production of starchy noodles, also called muk in Korea. Oligosaccharides, including raffinose, stachyose, and verbascose, in raw or poorly processed legumes are associated with flatulence in the human diet. While these oligosaccharides are present in mung beans, they are soluble in water and can be eliminated by adequate presoaking, germination, or fermentation. The energy offered by mung beans and sprouts is lower than that of other cereals, which is beneficial for individuals with obesity and diabetes [16]. In addition, trypsin inhibitors, hemagglutinin, tannins, and phytic acid found in the mung bean have also been reported to have biological functions, promoting digestion and eliminating toxins [17].

In addition to high protein and low energy content, mung beans also contain various enzymes and plentiful microelements. For example, superoxide dismutase (SOD) extracted from the mung bean can be chemically modified and made into an SOD oral liquid. This chemically modified SOD can avoid destruction by gastric acid and pepsin, thereby extending its half-life, making it suitable for human oral absorption [17].

Overall, regular consumption of mung beans could regulate the flora of enterobacteria, decrease the absorption of toxic substances, reduce the risk of hypercholesterolemia and coronary heart disease, and prevent cancer [18].

### Chemical constituents

**Figure 1:**
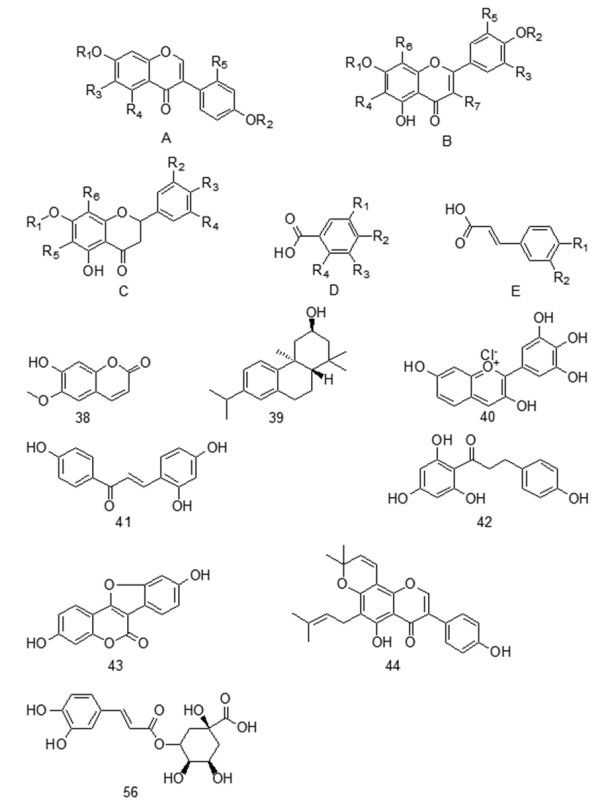
Structures of chemical components of mung bean seeds and sprouts.

During the past few decades, flavonoids, phenolic acids, organic acids and lipids have been identified from the seeds and sprouts of mung beans and have been shown to contribute to its pharmaceutical activities. The structures of these constituents and corresponding plant sources are summarized in Figure 1.

### Flavonoids

Flavone, isoflavone, flavonoids, and isoflavonoids (compounds **1–44** in Table 1) are the important metabolites found in the mung bean [19,20]. Most flavonoids have polyhydroxy substitutions and can be classified as polyphenols with obvious antioxidant activity. Vitexin (apigenin-8-C-*β*-glucopyranoside) and isovitexin (apigenin-6-C-*β*-glucopyranoside) have been reported to be present in mung bean seeds at about 51.1 and 51.7 mg g^−1^, respectively [21,22]. Flavonoids are involved in stress protection (i.e., oxidative and temperature stress), early plant development, signaling (i.e., legume nodulation), and protection from insect and mammalian herbivores [23].

**Table 1:** Chemical constituents identified from mung bean seeds and sprouts

**Flavonoids**										
**No.**	**Compound name**	**Skeleton**	**R1**	**R2**	**R3**	**R4**	**R5**	**R6**	**R7**	**Ref**
1	Daidzin	A	Glc	H	H	H	H	-	-	17
2	Daizein	A	H	H	H	H	H	-	-	17
3	Ononin	A	Glc	CH_3_	H	H	H	-	-	17
4	Formononetin	A	CH_3_	H	H	H	H	-	-	17
5	Isoformononetin	A	H	CH_3_	H	H	H	-	-	17
6	6,7,4′-trihydroxyisoflavone	A	H	H	OH	H	H	-	-	17
7	6,7,4′-trimethoxyisoflavone	A	CH_3_	CH_3_	OCH_3_	H	H	-	-	17
8	Genistin	A	Glc	H	H	OH	H	-	-	17
9	Sissotrin	A	Glc	CH_3_	H	OH	H	-	-	17
10	Genistein	A	H	H	H	OH	H	-	-	17
11	Prunetin	A	CH_3_	H	H	OH	H	-	-	17
12	Biochanin A	A	H	CH_3_	H	OH	H	-	-	17
13	6′′–O-acetylgenistin	A	GlcA	H	H	OH	H	-	-	17
14	5,7,4′-trimethoxyisoflavone	A	CH_3_	CH_3_	H	OCH_3_	H	-	-	17
15	2′-hydroxygenistein	A	H	H	H	OH	OH	-	-	17
16	Apigenin	B	H	H	H	H	H	H	H	17
17	Vitexin	B	H	H	H	H	H	Glc	H	20,21
18	Isovitexin	B	H	H	H	Glc	H	H	H	20,21
19	Rutin	B	H	H	H	H	OH	H	OGlc^6_^Rha	17
20	Quercetin-3-glucoside	B	H	H	OH	H	H	H	OGlc	17
21	Quercetin	B	H	H	OH	H	H	H	OH	17
22	Kaempferol	B	H	H	H	H	H	H	OH	17
23	Myricetin	B	H	H	OH	H	OH	H	OH	19
24	Rhamnetin	B	CH_3_	H	OH	H	H	H	OH	19
25	Kaempferitrin	B	Rha	H	H	H	H	H	ORha	19
26	Kaempferol-3-rutinoside	B	H	H	H	H	H	H	OGlc^6_^Rha	19
27	3,5,7,3′,4′-pentahydroxyflavonol	B	H	H	H	OH	OH	H	OH	19
28	3,7,8,3′,4′ -pentahydroxyflavonol	B	H	H	H	H	OH	OH	OH	19
29	Naringenin-7-glucoside	C	Glc	H	OH	H	H	H	-	17
30	Naringin	C	Glc^2_^Rha	H	OH	H	H	H	-	17
31	Neohesperidin	C	Glc^2_^Rha	H	OCH_3_	OH	H	H	-	18
32	Hesperetin	C	H	OH	OCH_3_	OGlc	H	H	-	18
33	5,7-dihydroxyflavanone	C	H	H	H	H	H	H	-	19
34	Eriodictyol-7-glucoside	C	Glc	H	OH	OH	H	H	-	19
35	Eriodictyol	C	H	OH	OH	H	H	H	-	19
36	Naringenin	C	H	H	OH	H	H	H	-	18
37	Rhododendrin	C	H	H	OH	H	CH_3_	CH_3_	-	19
38	Scopoletin	17								
39	Pomiferin	17								
40	Delphinidin	17								
41	2′,4,4′-trihydroxychalcone	17								
42	phloretin	17								
43	coumestrol	17								
44	osajin	17								
**Phenolic acids**										
45	*p*-hydroxybenzoic	D	H	OH	H	H	-	-	-	25
46	Protocatechuic	D	H	OH	OH	H	-	-	-	25
47	Syringic	D	OCH_3_	OH	OCH_3_	H	-	-	-	25
48	Gallic acid	D	OH	OH	OH	H	-	-	-	25
49	Vanillic acid	D	H	OH	OCH_3_	H	-	-	-	26
50	Gentisic acid	D	OH	H	H	OH	-	-	-	26
51	Shikimic acid	D	OH	OH	OH	H	-	-	-	26
52	*p*-coumaric	E	OH	H	-	-	-	-	-	26
53	Cinnamic acid	E	H	H	-	-	-	-	-	26
54	Caffeic acid	E	OH	OH	-	-	-	-	-	26
55	Ferulic	E	OH	OCH_3_	-	-	-	-	-	25
56	Chlorogenic acid									25

### Phenolic acids

Phenolic acids are secondary metabolites primarily synthesized through the pentose phosphate pathway (PPP) and shikimate and phenylpropanoid pathways [6]. Phenolic acids are major bioactive phytochemicals, and their presence in wild plants has facilitated the trend toward the increasing use of wild plants as foods [24,25].

Twelve phenolic acids (compounds **45–56** in Table 1) have been identified from mung bean seeds and sprouts [26,27]. Based on high levels of total phenolics and total flavonoids, mung beans show the benefits of 1,1-diphenyl-2-picrylhydrazyl (DPPH) radical scavenging activities, tyrosinase inhibition, and antiproliferative and alcohol dehydrogenase activities, which allow it to be used as a substitution for proper prescription drugs and as a preventative or therapeutic agent for the treatment of human diseases [28].

### Others

Organic acids and lipids have also been found in mung beans and sprouts. Twenty-one organic acids, including phosphoric and citric acid, and 16 lipids, including *γ*-tocopherol, were reported to be the major components of mung beans by gas chromatography/mass spectrometry (GC/MS) [29].

### Dynamic changes in metabolites

Under biotic and abiotic stress, plant physiology dramatically changes. The induction of defense systems, such as those involving proteinase inhibitors, produces a response that protects the plant from these types of stresses [30]. As a part of this response, accumulation of secondary metabolites with various health benefits has been observed [29,31]. However, in the absence of stress, healthy plants can also be stimulated by stress inducers to artificially produce secondary metabolites. Targeted analyses have demonstrated that the germination of mung beans is accompanied by a spectrum of significant changes in metabolite contents, such as decreased antinutrient concentrations [32] and increased levels of free amino acids [15,32-35].

Germination significantly reduces the levels of reducing sugars and starches by 36.1% and 8.78%, respectively [15]. Interestingly, until 60 h of incubation, levels of the monosaccharides fructose and glucose increase dramatically in the germinating material. However, significant reductions in the levels of both sugars have been observed during the final germination stage from 60 to 75 h. The concentration of the disaccharide sucrose increases within the first 24 h, but rapidly declines after the initial germination phase [9,15,29]. Moreover, raffinose and stachyose are completely eliminated during germination. The decline of sucrose in the latter stages of sprouting may be due to the lack of raffinose, resulting in the hydrolysis of sucrose for the energy supply [15].

Compared to cereals, mung beans contain higher amounts of protein [35]. As described earlier, proteolytic cleavage of proteins during sprouting leads to a significant increase in the levels of most amino acids. Additionally, increased levels of free amino acids in germinated mung beans and lentils have been observed via targeted analysis [33,36].

Gentistic acid, cinnamic acid, and *p*-hydroxybenzoic acid are the major phenolic acids of metabolites that are found throughout the sprouting process [37]. Within the first day of incubation, the levels of caffeic acid, ferulic acid, and shikimic acid are relatively low in mung bean seeds. However, after the initial soaking and early germination phase, mung bean samples exhibit significantly increasing amounts of these compounds [25]. Moreover, the levels of gallic acid, chlorogenic acid, and coumarin increase dramatically in the germination material until day 3 or 4, and catechin levels increase during the final stage of mung bean sprout development (i.e., on the eighth day of incubation) [26].

The overall levels of organic acids also increase during sprouting. Phosphoric and citric acid are 2 of the major organic acid metabolites. A distinct and continuous increase in lactic acid is observed, while malic acid and citric acid peak after only 24 h of incubation [29].

Fatty acid methyl esters (FAMEs) are formed mainly from transesterification of the crude lipid extract and reflect the presence of mung bean triglycerides. Within the first 24 h of incubation, changes in the levels of most FAMEs are relatively minimal. However, after the initial soaking and early germination phase, mung bean samples exhibit significant decreases in the levels of FAMEs. In contrast, the levels of *γ*-aminobutyric acid in mung bean sprouts are enhanced throughout sprout development and may be of special interest for human nutrition because of its health-promoting effects [29,38].

Protease inhibitors are proteins or peptides capable of inhibiting catalytic activities of proteolytic enzymes that play essential roles in biological systems, regulating proteolytic processes, and participate in defense mechanisms against a large number of insects, fungi, and other pathogenic microorganisms [39]. During the first 5 days of germination, there is a gradual decrease in the levels of extractable trypsin inhibitors in mung bean seeds [40]. The hemagglutinin activity of mung bean seeds has also been reported to decrease by about 84.4% after 3 days of germination [41].

### Biological activities

In ancient books, mung beans were well known for their detoxification activities. Mung bean protein, tannin, and other polyphenols are thought to combine with organophosphorus pesticides, mercury, arsenic, and other heavy metals, promoting the excretion of sediments from the body [42]. Mung beans have been shown to possess antioxidant, antimicrobial, and anti-inflammatory activities. Moreover, mung beans have antidiabetic, antihypertensive, lipid metabolism accommodation, antihypertensive, and antitumor effects, among others (Table 2). These various properties of this functional legume are discussed below.

**Table 2:** Biological activities and compounds of mung beans

**Biological activities**	**Biological compounds**
Antioxidant effects	Proteins, polypeptides, polysaccharides, polyphenols
Antimicrobial activity	Enzymes, peptides, polyphenols
Anti-inflammatory activity	Polyphenols
Antidiabetic effects	Polyphenols
Lipid metabolism accommodation	Phytosterol
Antihypertensive effects	Proteins, amino acids
Antitumor effects	Polyphenols, mung bean trypsin inhibitor fragments
Antisepsis effects	Polyphenols, aqueous extracts from mung bean coat

### Antioxidant effects

The proteins, polypeptides, polysaccharides, and polyphenols from the seeds, sprouts, and hulls of mung beans all show potential antioxidant activity. The antioxidant capacities of mung bean protein hydrolysate (MPH) have been reported as 0.67 and 0.46 μmol Trolox equivalent (TE)/mg protein, as measured by oxygen radical absorbance capacity-fluorescein (ORAC_FL_) and Trolox equivalent antioxidant capacity (TEAC) assays, respectively. Freeze-drying in lactose excipient reduces the antioxidant capacity of MPH to 0.48 μmol TE/mg protein in the ORAC_FL_ assay, but does not alter the results of the TEAC assay [43].

MP1 and MP2, isolated from the water extract of mung beans, are 2 acid heteropolysaccharides with 9.9% and 36.4% uronic acid content, respectively. The main composition of MP1 (molecular weight: 83 kDa) is mannose, whereas MP2 (molecular weight: 45 kDa) consists of rhamnose and galactose. MP2 exhibits higher hydroxyl radical-scavenging activity, while MP1 has higher reducing power and stronger scavenging capacity for superoxide and DPPH radicals, as well as greater inhibition of the self-oxidation of 1,2,3-phentriol than MP2 [44].

Importantly, mung bean extracts possess significantly higher radical scavenging activities, greater reducing power, and higher levels of polyphenols than soy bean extracts, suggesting that they are superior functional foods. Indeed, the radical scavenging activities of DPPH and 2,2′-azino-di-(3-ethyl-2,3-dihydrobenzthiazoline −6-sulfonate) (ABTS) isolated from mung bean extracts were found to be 11.33 ± 0.24 and 36.65 ± 0.63 μmol/g, respectively, and the ferric reducing antioxidant power (FRAP) of mung bean extracts was 31.85 ± 3.03 μmol/g. Mung bean extracts reduce the rate of pyrogallol autoxidation by 85% compared to the control and possess SOD-like activity of 83.48% ± 0.88% [45].

During the sprouting process, sprout extracts show higher amounts of total phenolics, total flavonoids, and DPPH radical scavenging activity than seed extracts [28]. Additionally, the antioxidant activity of mung bean sprouts is the highest on day 1 or 2, depending on the analysis method used (i.e., *β-*carotene assay or DPPH assay, respectively) [6].

The DPPH scavenging activity (SA) of mung bean soup (MBS; 20 mg/mL) is approximately 145% that of tea soup (5 mg/mL) and 195% that of vitamin C solution (0.15 mg/mL), indicating that the DPPH-SA of 100 g mung bean is equivalent to that of 36.3 g dried green tea and 1462 mg vitamin C. Vitexin and isovitexin are the major antioxidant components in mung beans [46]. Vitexin inhibits DPPH radicals by approximately 60% at 100 μg/mL and effectively prevents UV-induced skin cell death [47].

### Antimicrobial activity

The use of phytochemicals as natural antimicrobial agents, commonly called ‘biocides’ is gaining popularity. Enzymes, peptides, and polyphenols extracted from mung beans have been shown to possess both antimicrobial and antifungal activities. Assays for antifungal activity are usually executed using the method of inhibition crescents, while assays for antimicrobial activity are performed using the deferred plate method or the agar-diffusion method [48,49].

A nonspecific lipid transfer peptide (nsLTP; molecular weight: 9.03 kDa) with antimicrobial and antifungal activity was isolated from mung bean seeds. Interestingly, nsLTP exerts antifungal effects on *Fusarium solani*, *F. oxysporum*, *Pythium aphanidermatum*, and *Sclerotium rolfsii* and antibacterial effects on *Staphylococcus aureus* but not *Salmonella typhimurium*[50].

Mungin, a novel cyclophilin-like antifungal protein isolated from mung bean seeds, possesses activity against the fungi *Rhizoctonia solani*, *Coprinus comatus*, *Mycosphaerella arachidicola*, *Botrytis cinerea*, and *F. oxysporum*. Mungin also exerts inhibitory activity against *α*- and *β*-glucosidases, suppressing [^3^H] thymidine in corporation by mouse splenocytes [51].

In 2005, a chitinase (30.8 kDa) with antifungal activity was isolated from mung bean seeds. The protein has a pI of 6.3, as determined by isoelectric focusing, and an estimated specific activity of 3.81 U/mg. The enzyme exhibits optimal activity at pH 5.4 and is stable from 40 to 50°C. Importantly, chitinase exerts antifungal activity on *R. solani*, *F. oxysporum*, *M. arachidicola*, *P. aphanidermatum*, and *S. rolfsii*[52]*.*

In addition to the above antimicrobial and antifungal effects, polyphenol extracts from mung bean sprouts have also been shown to have activity against *Helicobacter pylori*, one of the most common bacterial infections in human beings causing gastroduodenal disease [6].

### Anti-inflammatory activity

In Asia, mung beans have been used in various cuisines and in folk remedies to treat toxic poisoning, heat stroke associated with thirst, irritability, and fever; these beneficial effects of mung beans are thought to be related to the inflammatory response [53].

Researchers have analyzed the anti-inflammatory effects of mung bean ethanol extracts on lipopolysaccharide (LPS)-stimulated macrophages. The extract mainly included polyphenols, gallic acid, vitexin, and isovitexin and markedly reduced the activity of murine macrophages through the prevention of pro-inflammatory gene expression without cytotoxicity [54]. Moreover, a study demonstrated that all pro-inflammatory cytokines, including interleukin (IL)-1*β*, IL-6, IL-12*β*, tumor necrosis factor (TNF)-*α*, and inducible NO synthase (iNOS), were dramatically down regulated in cells treated with 3.7 mg/mL polyphenols. These results suggested that the ethanol extract had great potential to improve the clinical symptoms of inflammation-associated diseases, such as allergies and diabetes [55].

The immune modulatory activities of mung bean water extracts and monomers on human peripheral blood mononuclear cells (PBMCs) have also been evaluated by BrdU immunoassay, secretion of interferon-gamma (IFN-*γ*) and IL-10, and elucidation of the responding cells by flow cytometry. The results demonstrated that 20 μg/mL genistein, phytic acid, and syringic acid induce a Th1-predominant immune response through significant suppression of IL-10 secretion and promotion of IFN-*γ* secretion. The study concluded that several non-nutritional ingredients of mung beans, such as flavonoids, acids, and plant hormones, are most likely to be important in the modulation of human immunity [56].

### Antidiabetic effects

Studies have also investigated the antidiabetic effects of mung bean extracts. In a study conducted in 2008, the antidiabetic effects of mung bean sprout extracts and mung bean seed coat extracts were investigated in type 2 diabetic mice (male KK-A^y^ mice and C57BL/6 mice). These extracts were orally administered to KK-A^y^ mice for 5 weeks, and mung bean sprout extracts (2 g/kg) and mung bean seed coat extracts (3 g/kg) lowered blood glucose, plasma C-peptide, glucagon, total cholesterol, triglycerides, and blood urea nitrogen (BUN) levels. At the same time, both treatments markedly improved glucose tolerance and increased insulin immunoreactive levels [57].

Phenolic antioxidants and levo-dihydroxy phenylalanine (L-DOPA) can be enriched in mung bean extracts through solid-state bioconversion (SSB) by *R. oligosporus*, with the goal of enhancing health-linked functionality. *α*-Amylase is responsible for cleaving starch during the digestive process, which is important in the management of postprandial blood glucose levels. A study in 2007 by Randir and Shetty investigated the inhibition of *α*-amylase and *H. pylori* in bioprocessed extracts and linked these effects to diabetes management and peptic ulcer management, respectively. The *α*-amylase inhibition potential of the tested sprouts extract was moderately high during early stages (days 0–2) and was higher during days 4–10, which correlated with higher phenolic content [58].

### Lipid metabolism accommodation

The modulation of lipid metabolism by mung bean has been well established. In an early study, rabbits with hyperlipidemia were fed a 70% mixture of mung bean meal and mung bean sprout powder. The mixtures affected the total cholesterol and *β*-lipoprotein content, alleviating symptoms of coronary artery diseases [59]. Additionally, in more recent studies, normal mice and rats were fed mung bean extracts for 7 days, and total cholesterol was significantly decreased in both types of rodents. This effect was thought to arise from the phytosterol content of mung beans, which was similar to blood cholesterol, facilitating the prevention of cholesterol biosynthesis and absorption [60].

### Antihypertensive effects

High doses (600 mg peptide/kg body weight) of raw sprout extracts, dried sprout extracts, and enzyme-digested sprout extracts have been shown to significantly reduce systolic blood pressure (SBP) in rats after administration for 6–9, 3–6, or 3–9 h, respectively. Similar changes were found in the plasma angiotensin I-converting enzyme (ACE) activity of these mung bean extracts. A long-term (1-month) intervention study that included treatment with fresh sprout powder, dried sprout powder, and concentrated extracts of the sprouts was carried out. The results indicated that the sprout powders were not as efficacious as concentrated sprout extracts. The SBPs of rats treated with concentrated extracts of fresh and dried sprouts were significantly reduced during the intervention period from weeks 1–4 and weeks 2–4, respectively [61].

### Antitumor effects

Mung beans have been shown to exert antitumor effects through several different mechanisms. The recombinant plant nucleases R-TBN1 and R-HBN1, similar to nucleases derived from pine pollen and mung beans, were found to be effective against melanoma tumors and were about 10-times more potent than bovine seminal ribonuclease (RNase). Due to their relatively low cytotoxicity and high efficiency, these recombinant plant nucleases appear to be stable biochemical agents that can be targeted as potential antitumor cytostatics [62].

In addition, mung beans have been shown to exert antiproliferative effects, as examined by MTT [3-(4,5-dimethylthiazol-2-yl)-2,5-diphenyltetrazolium bromide] assay using an *in vitro* cell culture system. Mung beans exhibit dose-dependent antiproliferative effects against the tongue squamous cell carcinoma cell line CAL27 and several other cancer cell lines tested (i.e., DU145, SK-OV-3, MCF-7, and HL-60 cells) [63].

Another study evaluated the effects of trypsin inhibitors from mung beans (i.e., LysGP33) on the metastasis and proliferation of human colon cancer cells (SW480 cells). In this study, the effects of the purified GST-LysGP33 active fragment on the migration of SW480 cells were detected using wound healing assays. The results showed that 10 μmol/L GST-LysGP33 active fragment affected cell migration beginning at the 24-h time point. After 72 h, cells treated with GST-LysGP33 exhibited an approximate 50% reduction in wound healing compared to the control group [64].

### Antisepsis effects

The aqueous extract from mung bean coat (MBC) is protective against sepsis *in vitro* and *in vivo*. The effect was achieved by the inhibition of high mobility group box 1 (HMGB1), a nucleosomal protein that has recently been established as a late mediator of lethal systemic inflammation with a relatively wider therapeutic window for pharmacological interventions. It was found that MBC dose-dependently attenuated the LPS-induced release of HMGB1 and several chemokines in macrophage cultures. The animal survival rates after oral administration of MBC were significantly increased from 29.4% (in the saline group, N = 17 mice) to 70% (in the experimental MBC extract group, N = 17 mice, *P* < 0.05) [65]. Chlorogenic acid (56) has also been shown to be protective against lethal sepsis by inhibiting late mediators of sepsis. Chlorogenic acid suppresses endotoxin-induced HMGB1 release in a concentration-dependent manner in murine peritoneal macrophages. Additionally, administrations of chlorogenic acid attenuate systemic HMGB1 accumulation *in vivo* and prevented mortality induced by endotoxemia and polymicrobial sepsis [66].

## Conclusion

The mung bean [*Vigna radiata* (L.) Wilczek] is one of the most important short-season, summer-growing legumes and is grown widely throughout tropic and subtropic regions. As we have discussed in this review, mung beans have wide applications in agriculture, health food, pharmaceutical, and cosmetics industries. Mung bean seeds and sprouts are excellent examples of functional foods that lower the risk of various diseases. Moreover, the seeds and sprouts have health-promoting effects in addition to their nutritive value.

During the germination process of the mung bean, its chemical constituents undergo a series of biochemical reactions. One such reaction is the synthesis of small active compounds from macromolecular substances, promoting absorption and utilization. Another change observed during germination is the formation and accumulation of many types of active substances, such as polyphenols, saponins, vitamin C, etc. Therefore, we believes that these changes in the chemical composition of mung beans during germination will lead to substantial and important changes in the pharmacological activities of mung beans as well.

Research into the chemical constituents and biological activities of mung bean seeds and sprouts have provided a solid theoretical basis for the development and utilization of mung beans. Combined with analysis of the metabolites of these chemical constituents, research investigating the physiological functions of these compounds is required for further advancement of this field. Thus, future studies may focus on the extraction and purification of new physiologically active substances in agriculture, health foods, cosmetics, and pharmaceutical applications.

## Abbreviations

FAO/WHO: Food and Agriculture Organization/World Health Organization; SOD: Superoxide dismutase; PPP: Pentose phosphate pathway; DPPH: 1,1-Diphenyl-2-picrylhydrazyl; GC/MS: Gas chromatography/mass spectrometry; FAMEs: Fatty acid methyl esters; MPH: Mung bean protein hydrolysate; TE: Trolox equivalent; ORACFL: Oxygen radical absorbance capacity-fluorescein; TEAC: Trolox equivalent antioxidant capacity; ABTS: 2,2′-Azino-di-(3-ethyl-2,3-dihydrobenzthiazoline −6-sulfonate); FRAP: Ferric reducing antioxidant power; SA: Scavenging activity; MBS: Mung bean soup; nsLTP: Nonspecific lipid transfer peptide; LPS: Lipopolysaccharide; IL: Interleukin; TNF: Tumor necrosis factor; PBMCs: Peripheral blood mononuclear cells; IFN-γ: Interferon-gamma; BUN: Blood urea nitrogen; L-DOPA: Levo-dihydroxy phenylalanine; SSB: Solid-state bioconversion; SBP: Systolic blood pressure; ACE: Angiotensin I-converting enzyme; RNase: Ribonuclease; MTT: [3-(4,5-dimethylthiazol-2-yl)-2,5-diphenyltetrazolium bromide]; MBC: Mung bean coat; HMGB1: High mobility group box 1.

## Competing interests

The authors declare that they have no competing interests.

## Authors' contributions

DY, LL, and RH were involved in preparing the manuscript. TD and HC participated in discussions of views represented in the paper. All authors have read and approved the final manuscript.
